# Efficacy of a Recombinant Turkey Herpesvirus AI (H5) Vaccine in Preventing Transmission of Heterologous Highly Pathogenic H5N8 Clade 2.3.4.4b Challenge Virus in Commercial Broilers and Layer Pullets

**DOI:** 10.1155/2018/3143189

**Published:** 2018-11-21

**Authors:** Vilmos Palya, Tímea Tatár-Kis, Edit Walkóné Kovács, István Kiss, Zalán Homonnay, Yannick Gardin, Krisztián Kertész, Ádám Dán

**Affiliations:** ^1^Scientific Support and Investigation Unit, Ceva-Phylaxia, Ceva Animal Health, Budapest 1107, Hungary; ^2^Ceva Animal Health, Libourne 33500, France; ^3^Ceva-Phylaxia, Ceva Animal Health, Budapest 1107, Hungary; ^4^Veterinary Diagnostic Directorate, National Food Chain Safety Office (NEBIH), Budapest 1149, Hungary

## Abstract

Outbreaks caused by the highly pathogenic avian influenza virus (HPAIV) H5N8 subtype clade 2.3.4.4 were first reported in 2014 in South Korea then spread very rapidly in Asia, to Europe, and for the first time, to North America. Efficacy of a recombinant HVT-AI (H5) vaccine (rHVT-H5) to provide clinical protection as well as to significantly reduce the shedding of an H5N8 challenge virus has already been demonstrated in SPF chickens. The aim of our studies was to test the efficacy of the same rHVT-H5 vaccine in controlling the transmission of a recent Hungarian HPAIV H5N8 challenge virus in commercial chickens. Broilers and layers were vaccinated at day old according to the manufacturer's recommendation and then challenged with a 2017 Hungarian HPAIV H5N8 (2.3.4.4b) isolate at 5 or 7 weeks of age, respectively. Evaluation of clinical protection, reduction of challenge virus shedding, and transmission to vaccinated contact birds was done on the basis of clinical signs/mortality, detection, and quantitation of challenge virus in oronasal and cloacal swabs (regularly between 1 and 14 days postchallenge). Measurement of seroconversion to AIV nucleoprotein was used as an indicator of infection and replication of challenge virus. Our results demonstrated that rHVT-H5 vaccination could prevent the development of clinical disease and suppress shedding very efficiently, resulting in the lack of challenge virus transmission to vaccinated contact chickens, regardless the type of birds. Single immunization with the tested rHVT-H5 vaccine proved to be effective to stop HPAIV H5N8 (2.3.4.4b) transmission within vaccinated poultry population under experimental conditions.

## 1. Introduction

In recent years, several reassortant H5Nx subtype of highly pathogenic avian influenza (HPAI) viruses have emerged in East Asia. These new viruses, mostly of subtype H5N1, H5N2, H5N6, and H5N8, belonging to clade 2.3.4.4, have spread very rapidly in East Asia causing outbreaks in poultry in China, South Korea, and Vietnam. Virus strains related to the Eurasian H5N8 lineage of clade 2.3.4.4 have also spread over considerable distances reaching Europe (2014-2015 and 2016-2017) and for the first time, the North American continent (2014-2015). This lineage of clade 2.3.4.4 which is circulating in wild bird populations regularly infects backyard poultry as point source of introductions to industrial poultry and has caused repeated epidemics in several parts of the world [1, 2].

In several countries, the outbreaks of HPAI have been controlled by rapid depopulation of infected poultry premises, preemptive culling of neighbouring farms, movement restrictions, and sanitary measures [3]; however, the application of this control method could have a devastating effect on the economy. The need for effective vaccines against HPAI has been arose by affected countries not only for the survival of the poultry industry but also because of the risk of future recurrence and persistence of the disease and its transmission potential to humans.

A number of H5 avian influenza vaccines, including the inactivated whole virus vaccines and live recombinant vaccines using fowlpox virus or turkey herpesvirus (HVT) or Newcastle disease virus (NDV) as vectors to express the HA antigen of a selected H5 subtype avian influenza virus (AIV) strain [4], are currently available for use in poultry. Traditional avian influenza (AI) vaccines are killed vaccines, produced either by conventional methods or by reverse genetics [5] which provide good protection against the clinical disease caused by HPAIVs and significant reduction in viral shedding, if the vaccine seed strain is antigenically matched to the challenge strain [6]. However, killed vaccines have several limitations including (i) the requirement for frequent update of vaccine seed strains to match with the circulating field strains, (ii) the interference of maternally derived antibodies (MDA) with vaccination, (iii) the lack of possibility to differentiate vaccinated birds from infected ones (DIVA) by serology unless the vaccine strain contains heterologous NA to all potentially circulating field viruses in the given geographical area/country, and (iv) the lack of stimulating strong cellular immunity (killed vaccines mostly stimulate a humoral immune response). Because of these shortcomings of killed vaccines, next generation technology has been used to develop a wide variety of AI vaccines to overcome some of these limitations [7].

HVT proved to be an excellent candidate for vector since it (i) confers long-term immunity due to its persistence in the host, (ii) has excellent safety characteristics, (iii) provides good protection when administered at hatch or in ovo, (iv) overcomes MDA, (v) can be used in validated combinations with certain other Marek's disease vaccines of other serotypes (e.g., [8, 9]), and (vi) may provide possibility to apply the DIVA strategy [10]. Attempts to use HVT as vector vaccine started in the early 1990s [11, 12]; however, it was not until more recently that HVT has been widely used as a vector for the development of recombinant vaccines against a number of poultry viral diseases, including the ones expressing AIV proteins for the protection against HPAI [13–16]. One of these candidate rHVT-AI vaccines has already reached marketing authorization in a number of countries and demonstrated promising results in poultry in several studies [17] including efficacy against H5Nx clade 2.3.4.4. isolates [18–20].

To asses the potential impact of control measures such as vaccination, it is crucial, however, to understand the transmission dynamics of AI virus both in susceptible and vaccinated populations. The potential of a vaccine to control the spread of infection at population level should be an important part of investigation when studying the effectiveness of a vaccine in the control of infectious diseases. Therefore, the goal of this study was not only to evaluate the efficacy of a commercial, live recombinant HVT-based AI vaccine against a recent H5N8 clade 2.3.4.4b virus in commercial broiler and layer chickens but to examine and quantify the effect of vaccination on virus transmission as well.

## 2. Materials and Methods

### 2.1. Vaccine

The commercial HVT vector-based live recombinant AI vaccine (Vectormune® AI, Ceva Biomune, Lenexa, KS) expressing the H5 gene (rHVT-H5) of a clade 2.2 H5N1 HPAIV was used in this study. Donor of the vaccine HA was the A/mute swan/Hungary/4999/2006 strain for which the cleavage site has been modified for a low pathogenic motif. The vaccine (lot number: 395-054) was diluted in the corresponding diluent (Ceva-Biomune, Lenexa, KS) to contain one dose in 200 *μ*l.

### 2.2. Challenge Virus

The A/goose/Hungary/1030/2017 H5N8 HPAIV (HA clade 2.3.4.4b), isolated during the recent 2016-2017 epidemic of HPAI in Hungary, obtained from the virus repository of National Food Chain Safety Office Veterinary Diagnostic Directorate (NFCSO-VDD), Budapest, Hungary, was used in this study. The virus was propagated and titrated in specific-pathogen-free (SPF) embryonated chicken eggs according to standard procedures [21]. Titer was calculated using Spearman-Kärber method [22].

### 2.3. Antigenic Relatedness of Challenge Virus with the Vaccine

#### 2.3.1. Comparison of Haemagglutinin Amino Acid Sequence of Vaccine Insert and Challenge Strain

The deduced amino acid sequences of challenge virus HA gene (accession number is EPI954663 in GISAID EpiFlu database) and the rHVT-H5 vaccine insert (accession number is KP969039 in GenBank) were aligned and pairwise comparison was prepared in CLC Main Workbench 7.9.1. Predicted H5 epitopes were annotated based on the identified epitopes by using polyclonal rabbit antisera for epitope scanning of baculovirus-expressed H5 HA protein [23]. Further antigenicity-associated sites [24, 25], predicted MHC antigenic sites [25, 26], and the previously identified MHCI/II peptide [27] were also compared between the vaccine and the challenge virus.

#### 2.3.2. One-Way Cross-Haemagglutination Inhibition Test

The antigenic relatedness between the vaccine and challenge virus was determined by measuring the haemagglutination inhibition (HI) titer of antisera raised against the vaccine virus using HA antigens homologous with the vaccine or with the challenge virus (see in Serology paragraph). The antisera were collected six weeks after vaccination of day-old SPF chicks with the rHVT-H5 vaccine.

### 2.4. Experimental Design

The broiler (breed: Ross 308) and layer (breed: Tetra-SL) chicks used in the two transmission experiments which are described in this paper were obtained from commercial sources in Hungary (from hatcheries of Herbro Kft, Hernád and Tetra Kft, and Bábolna, respectively). Serum samples were collected at hatch from 10 individuals of each type of chicks to ascertain that the birds were serologically negative for antibodies to the nucleoprotein of influenza A viruses. During the postvaccination/prechallenge period, chickens were housed in BSL-2 animal facilities and then transferred to BSL3 animal rooms for challenge (3 chickens/m^2^). In both cases, chickens were kept on deep litter and water was provided through nipple drinkers or drinking towers which were controlled and changed daily. Appropriate food was provided *ad libitum*. All animals were housed separately according to group (i.e., one group in each room) in isolated animal rooms at Prophyl Kft. (Bar, Hungary).

The study has been conducted in compliance with the provisions of Directive 2010/63/EU, Hungarian Act No. XXVIII/1998, and the Hungarian Governmental Decree No. 40/2013 (II.14.) and with the permission of the Hungarian competent animal welfare and ethics authority (approval number: BAI/35/56-92/2017). No humane endpoint was used, since the study aimed at measuring the transmission rate of HPAIV and its outcome would be biased by the earlier removal of strong shedder diseased chickens. Generally, the birds died after short clinical phase (no clinical signs were observed on the day preceeding mortality in 22% of chickens; clinical signs were observed for less than 24 hours in 45%, for less than 48 hours in 26%, and for less than 72 hours in 7% of chickens). At the end of postchallenge observation period, all survived chickens were euthanized by injection of sodium pentobarbital (5 g/ml).

#### 2.4.1. Transmission Experiment 1: Broilers

Two groups (groups 1 and 2) of broiler chicks were used (see Table 1). Each group consisted of 40, one-day-old chicks. 40 chicks of group 1 were vaccinated (designated G1-V for vaccinated) while the chicks of group 2 remained unvaccinated (designated G2-S for susceptible). All chicks of group 1 were vaccinated with a commercial dose of the rHVT-H5 vaccine subcutaneously (s.c.) on the nape of the neck, using a needle of 19G × 1″ at one day of age. The two groups were housed separately and were checked daily.

On day 31 postvaccination (p.v.), blood samples were taken from each animal for serology. Sera were separated from the blood clots by centrifugation, then inactivated at 56°C for 30 min, and stored at −20°C. On day 36 p.v., 20 animals from both the vaccinated (designated G1-VCh for vaccinated direct challenged) and from the nonvaccinated group (designated G2-SCh for susceptible direct challenged) were tranferred to separate BSL3 animal rooms and were challenged by inoculating them intranasally with 10^6^ ELD_50_/0.2 ml of the H5N8 virus. Eight hours postchallenge (pch.), the remaining 20 animals from each of the relevant groups were added as contact birds (designated G1-VC for vaccinated contact or G2-SC for susceptible contact).

The animals were observed for 14 days after challenge during which they were checked twice daily for clinical signs and mortality. Oronasal (ON) swabs taken from the choanal slit and cloacal (CL) swabs using Copan FLOQSwabs™ (ref 552C, Copan Diagnostics Inc., CA, USA) were collected daily between day 1 and 7 and then at day 10 pch. From the animals found dead, oronasal and cloacal swabs were also collected. In case the diagnosis of HPAI was not unambiguous based on the clinical signs and the gross lesions, organ samples (brain, heart, kidney, and spleen) were collected for qRRT-PCR. Those chickens, in which the cause of mortality was not attributable to challenge based on these laboratory tests, were omitted from the evaluation of clinical protection. At the end of the experiment (at 14 days pch., 50 days of age), oronasal and cloaca swabs as well as 5 ml of blood were collected from all surviving animals, and then the chickens were euthanized.

#### 2.4.2. Transmission Experiment 2: Layers

In this experiment, two groups (groups 3 and 4), each consisting of 40, one-day-old layer chicks, were used (see Table 1). Chicks of group 3 were vaccinated (designated G3-V), while the chicks of group 4 remained unvaccinated (designated G4-S for susceptible). For vaccination, the same procedure as described for transmission experiment 1 was followed. The animals were housed in two rooms and were treated as described for transmission experiment 1. The chickens were blood sampled on day 21, 28, 35, and 45 p.v. On day 50 p.v., 20 animals from both the vaccinated (designated G3-VCh for vaccinated direct challenged) and from the nonvaccinated group (designated G4-SCh for susceptible direct challenged) were tranferred to separate BSL3 animal rooms and then challenged as described in transmission experiment 1. Sampling and euthanasia protocols remained the same as described in transmission experiment 1. The experiment was terminated at 14 days pch. (i.e., 9 weeks of age).

### 2.5. Detection of rHVT-H5 Vaccine from Feather Pulp

To assess the take of rHVT-H5 vaccine (replication of vaccine virus in the bird), feather pulp samples were collected from five chickens of each vaccinated group at 3 weeks of age and from 5 vaccinated broilers and 10 vaccinated layers at 4 weeks of age. Five nonvaccinated animals were sampled the same way. Feather tips with substantial amount of pulp were homogenized in 1 ml phosphate-buffered saline by using Tissue Lyser II (Qiagen, Hilden, Germany). After centrifugation at 1500 × g at 4°C for 10 min, the supernatant of samples was processed as described previously [28].

### 2.6. Serology

Hemagglutination inhibition (HI) test performed according to standard procedure (OIE) was used to determine antibody responses elicited by vaccination and to check serological relatedness between the vaccine virus and the challenge virus. Two antigens have been used. One of them was closely related to the insert of the vaccine and considered as homologous antigen with the vaccine (reverse genetics antigen containing HA from clade 2.2.1 virus; Rg-A/duck/Egypt/M2583/10 (dH5N1)-A/PR/8/34/(R) (6 + 2), [29]) and the other one was prepared from the H5N8 challenge virus propagated in SPF hen eggs according to standard procedures [21] and then inactivated (Veterinary Diagnostic Directorate, National Food Chain Safety Office, Budapest, Hungary). Antiserum to the rHVT-H5 vaccine prepared in SPF chickens and serum samples collected in both transmission experiments before the challenge were checked against 4 HAU both of the antigen homologous to the vaccine and the antigen homologous to the challenge strain in order to evaluate cross-reactivity of vaccine-induced antibodies with the challenge virus. HI titers are reported as log_2_ values, with 3 log_2_ being the minimum titer considered as positive. Serum samples with HI titer below 1 : 2 were included with 0 log_2_ HI titer in the calculation of mean titer. Log_2_ titers obtained with the two different antigens from the same serum samples were compared with paired *t*-test at 95% confidence level.

The ID Screen® Influenza A Nucleoprotein Indirect ELISA kit (for the specific detection of nucleoprotein antibodies; code: FLUNPS, IDVet, France) was used according to the manufacturer's instructions. Only the serum samples collected at day old to check the absence of MDA to AIV and serum samples collected at the termination of the experiments (14 days pch.) to check if the challenge caused infection and induction of antibodies to the nucleoprotein (NP) of AIV (DIVA) were tested by ELISA.

### 2.7. Challenge Virus RNA Quantification from Oronasal and Cloacal Swabs

To measure the number of challenge virus RNA copies present in the dry swabs collected during the transmission experiments, quantitative real-time reverse transcriptase PCR (qRRT-PCR) was used. After elution in 2 ml of PBS, 200 *μ*l of swab supernatant was submitted to RNA extraction (MagNA Pure LC DNA and viral NA small volume nucleic acid isolation kit on the MagNA Pure LC robotic instrument (Roche Diagnostics, Indianapolis, IN)) according to the manufacturer's instructions. The extracted RNA was eluted in 100 *μ*l elution buffer.

Real-time RT-PCR reactions were performed according to the EU diagnostic manual (2006) with protocol developed and validated at the European Reference Laboratory for AIV/NDV (Animal Health and Veterinary Laboratories Agency, Weybridge, UK) using the general influenza A primers and probe for matrix protein gene (M-gene) and one-step RT-PCR kit (Qiagen, Germany) as originally described by Spackman et al. [30].

All oronasal or cloaca swab samples which gave specific signal were considered positive regardless the Ct value. Standard curve was established with viral RNA extracted from serial dilution of the titrated challenge virus suspension, and AIV load in ELD_50_/ml was calculated by extrapolation of Ct values of swab samples to this standard curve. The lowest limit of detection based on the swab samples from chickens was 10^2.1^ELD_50_/ml. All negative samples were included in the calculation of mean with a value of 10^1.5^ELD_50_/ml.

### 2.8. Statistical Analysis of Challenge Virus Transmission (Calculation of R)

For the evaluation of challenge virus transmission, a bird was considered infectious at a certain sampling date if at least one of its swab samples (oronasal or cloaca) was positive. Virus transmission rate was estimated by the susceptible-infectious-recovered (SIR) stochastic model, using generalized linear models (GLM). The formulation of the transmission model is as follows. We accepted that the number of new cases (C (t)) is binomially distributed:

(1) $$\begin{array}{c} C\left(t\right)\text{\textasciitilde{}}Binomial\left[S\left(t\right),p\left(\Delta \left(t\right)\right)\right], \end{array}$$

where

(2) $$\begin{array}{c} p\left(\Delta \left(t\right)\right)=1-\exp\left(-\beta \frac{I\left(t\right)}{N\left(t\right)}\Delta \left(t\right)\right). \end{array}$$

*S* (*t*) is the number of susceptible birds at beginning of day *t*, *I* (*t*) is the number of infectious chickens at beginning of day *t*, *N* (*t*) is the number of live chickens at the beginning of day *t* (animals that died were removed from the day of death), Δ (*t*) is the number of days elapsed between day *t* and day *t* − 1, and *β* is the transmission rate parameter to be estimated.

The above model can be formulated as a GLM with a complementary log-log link, taking log((*I*(*t*)/*N*(*t*))Δ(*t*)) as offset variable. The intercept of GLM estimate is

(3) $$\begin{array}{c} \log\left(\beta \right). \end{array}$$

The mean infectious period (*T*) was directly calculated from the data. For this, we used the data of the contact animals only as we tried to model the transmission in field conditions.

The reproduction ratio is given by the product of the transmission rate and the mean infectious period:

(4) $$\begin{array}{c} R=\beta T. \end{array}$$

For the calculation of the confidence interval of *R*, we assumed independence between log (*β*) and log (*T*). Thus, by using the delta method to estimate Var (log(*T*))

(5) $$\begin{array}{c} Var\left(\log\left(R\right)\right)=Var\left(\log\left(\beta \right)\right)+Var\left(\log\left(T\right)\right)=Var\left(\log\left(\beta \right)\right)+\frac{Var\left(T\right)}{T^{2}} \end{array}$$

and the 95% confidence interval for *R* is

(6) $$\begin{array}{c} \exp\left[\log\left(R\right)\pm 2\cdot \sqrt{Var\left(\log\left(R\right)\right)}\right]. \end{array}$$

Statistical computations were performed using Stata software.

## 3. Results

### 3.1. Antigenic Relatedness between the Challenge Virus and the Vaccine

#### 3.1.1. Sequence Comparison of rHVT-H5 Vaccine Insert and Challenge Virus HA Gene

Amino acid identity was 92%, which meant 45 amino acid differences between the sequences, including a single amino acid deletion from the sequence adjacent to the cleavage site, i.e., 337PQGERRRKKR/G347 and 337PLREKRRKR/G347 for the rHVT-H5 vaccine insert and for the challenge virus strain, respectively.

**Figure 1:**
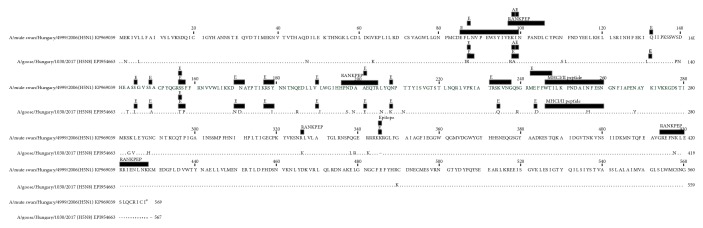
Alignment of A/mute swan/Hungary/4999/2006 (H5N1) and A/goose/Hungary/1030/2017 (H5N8) HA amino acid sequences. Identical residues are marked by dots. The sites of antigenic relevance (abbreviation: “A”), the predicted epitopes (abbreviation: “E”), the predicted MHC epitopes by RANKPEP, and the MHCI/II epitope identified for AIV H5 are marked by shaded boxes according to published reports [23–25, 27].

Several of the predicted epitopes or antigenic sites were affected by substitutions in the challenge virus compared to the rHVT-H5 vaccine insert sequence (see Figure 1). Haghighi et al. identified an MHCI/II peptide in the HA of A/turkey/Ireland/1378/83 (H5N8) which was able to stimulate both CD4+ and CD8+ lymphocytes [27]. This peptide motif (H5_246–260_) can be found in the HA of both the vaccine and the challenge virus and shows a single amino acid difference between them at residue 256 being asparagine (N) in the vaccine and histidine (H) in the challenge virus.

#### 3.1.2. Cross-Haemagglutination Inhibition Test

Testing of antisera prepared against the rHVT-H5 vaccine in SPF chickens resulted in 5.4 ± 0.6 log_2_ HI titer (mean and SD) with the antigen homologous to the vaccine, while only 0.3 ± 1.0 log_2_ HI titer (mean and SD) was measured with the challenge virus antigen indicating strong antigenic distance between the vaccine and the challenge virus.

### 3.2. Testing Day-Old Serum Samples from Commercial Broilers and Layers for MDA to AIV

ELISA detecting antibodies against nucleoprotein of influenza A virus was used to prove the negativity of the test chickens for AIV antibodies. Blood samples collected from both the broiler and layer chicks at one day of age were negative for AIV antibodies.

### 3.3. Vaccine Take and Antibody Response to Vaccination in Broilers and Layers without MDA to AIV

**Figure 2:**
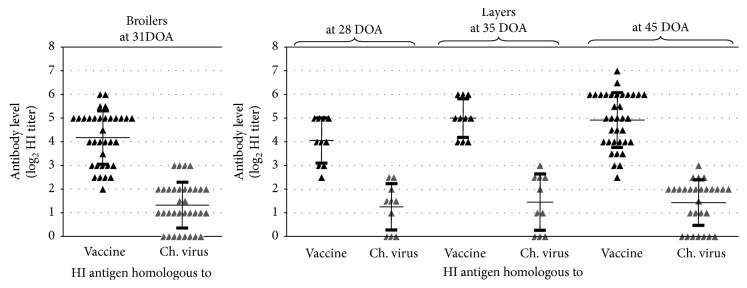
Humoral immune response to vaccination in commercial broilers and commercial layers without MDA to AIV H5N1. Hemagglutination inhibition test was performed parallel with the antigen homologous to the vaccine and with the antigen prepared from the challenge virus. Age of birds at sampling is shown above the graph (DOA = days of age). Mean titer and standard deviation is shown as a horizontal bar with whiskers. Positivity limit is at least 3 log_2_ HI titer.

Vaccine virus was detected from the feather tips of all vaccinated chickens at 3 weeks of age, while 80% positivity was found at 4 weeks of age (4/5 positives in broilers and 8/10 positives in layers). Humoral immune response to rHVT-H5 vaccination was tested by HI test against HA antigens homologous either with the vaccine virus insert or with the challenge virus. All chickens, both in the broiler and layer experiments, were positive by HI test from four weeks of age onwards using the vaccine homologous HA antigen. On the contrary, the group mean HI titers measured against the challenge virus remained below the positivity threshold (see Figure 2) and were significantly lower compared to the titers measured with HA antigen homologous with the vaccine (*p* ≤ 0.001). The nonvaccinated animals remained seronegative during the whole observation period before the challenge (all serum samples with HI titer below 1 : 2).

### 3.4. Transmission Experiment 1: Broilers

#### 3.4.1. Clinical Signs and Mortality

**Figure 3:**
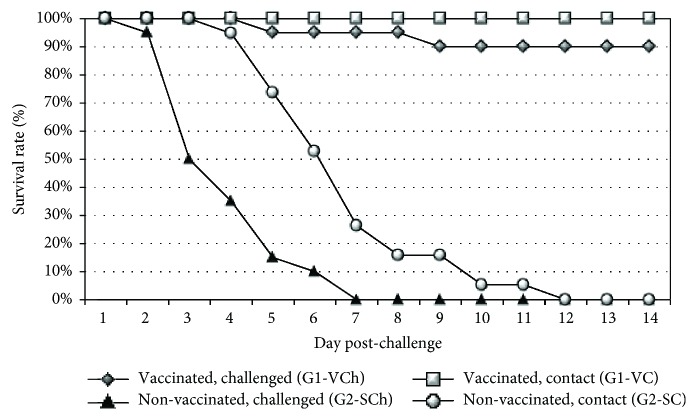
Time course of mortality in challenged broilers. Contact challenged groups were comingled with direct-challenged groups from 8 hours postchallenge. Recording date refers to the time after direct challenge.

The H5N8 challenge virus, consistent with the highly pathogenic nature of this virus strain, was lethal for the nonvaccinated broiler chickens. After challenge infection, all animals both in the inoculated and contact groups (G2-SCh and G2-SC) developed clinical signs indicative of HPAI which was followed by 100% mortality in the direct challenged and in the contact group too (see Figure 3). The most prominent clinical signs in the nonvaccinated challenged broiler chickens were lethargy, anorexia, prostration, and neurologic signs.

By contrast, 90 percent of vaccinated chickens were protected against the HPAIV challenge and none of the vaccinated contact chickens died or showed clinical signs indicative of HPAIV infection during the postchallenge observation period (see Figure 3). Unfortunatelly, two vaccinated contacts died due to accidental physical injury (organ and swab samples collected from them proved to be negative with qRRT-PCR).

#### 3.4.2. Virus Shedding and Transmission

**Figure 4:**
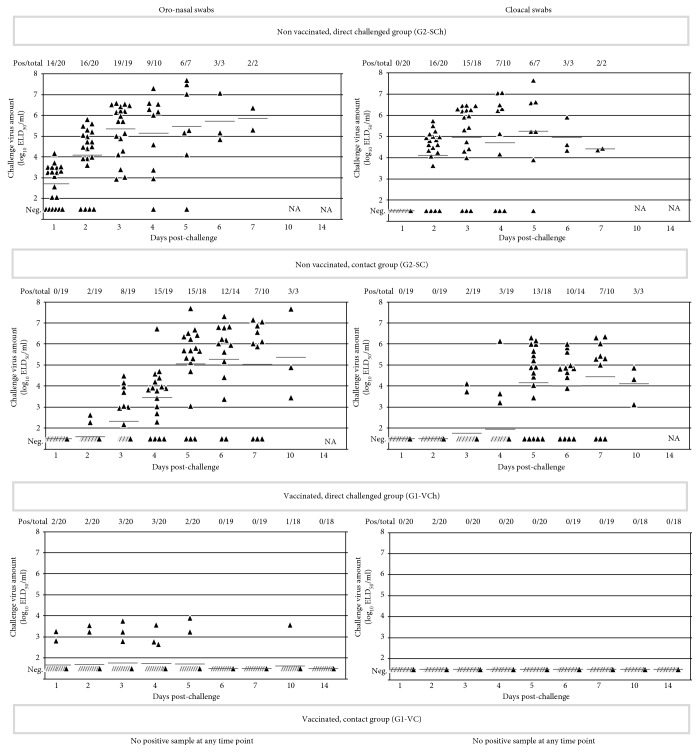
Oronasal and cloacal shedding by broilers. AIV load of swab samples measured by qRRT-PCR is presented as log_10_ELD_50_/ml in the scatterplot; horizontal bars represent group mean value for each date. For calculation of mean, negative samples were given a value of 1.5 log_10_ ELD_50_/ml. Contact challenged groups (G1-VC and G2-SC) were comingled with the direct-challenged control group (G1-VCh and G2-SCh) from 8 hours postchallenge. Sampling date refers to the time after direct challenge.

In the nonvaccinated group, all of the direct challenged and contact animals shed high amount of challenge virus by the oronasal route (see Figure 4). After challenge, fast increasing virus load was measured in the direct inoculated birds, and similar, but slightly slower increase of virus shedding could be observed in the contact chickens 3-4 days later.

Fast increasing, high virus load was measured in the cloacal swabs of the nonvaccinated direct-challenge birds that was followed a few days later in the contact chickens reaching similar virus loads to the direct-challenged animals.

The majority of direct-challenged vaccinated chickens were negative by qRRT-PCR in ON swabs. Virus shedding could be detected at low level in ten percent of chickens mainly during the first 5 days pch. Only one inoculated animal was found to be qRRT-PCR positive for the challenge virus in its ON swab with low virus load on day 10 pch. (see Figure 4). No virus shedding could be detected during the observation period in the vaccinated contact chickens (G1-VC).

A graphic representation of the progress of infection in the directly challenged and contact animals for the vaccinated groups for each day pch. is shown in Figure 4.

The challenge virus could not be detected in the cloacal swabs of vaccinated chickens, except one chicken with moderate level of shedding at day 6 pch. All vaccinated contact chickens had negative cloaca swab samples during the observation period.

### 3.5. Transmission Experiment 2: Layers

#### 3.5.1. Clinical Signs and Mortality

**Figure 5:**
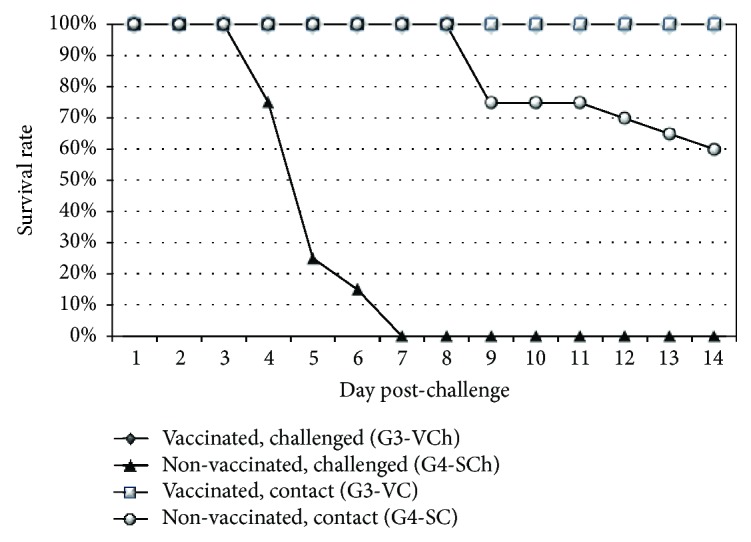
Time course of mortality in challenged layers. Contact challenged groups were comingled with direct-challenged groups from 8 hours postchallenge. Sampling date refers to the time after direct challenge.

All of the unvaccinated direct-challenged birds (designated G4-SCh) died by 7 days pch. Spreading of challenge virus was slower in layer chickens than in broilers; morbidity of the nonvaccinated contacts (designated G4-SC) began five days later than in the direct-challenged chickens. Delayed infection of contact chickens resulted in lower morbidity and mortality (40%) compared to direct-challenged layers by the end of the postchallenge observation period (see Figure 5).

No mortality or clinical signs indicative of HPAIV infection occurred either in the vaccinated direct-challenged group (G3-VCh) or in the vaccinated contact chickens (G3-VC) during the postchallenge observation period (see Figure 5).

#### 3.5.2. Virus Shedding and Transmission

**Figure 6:**
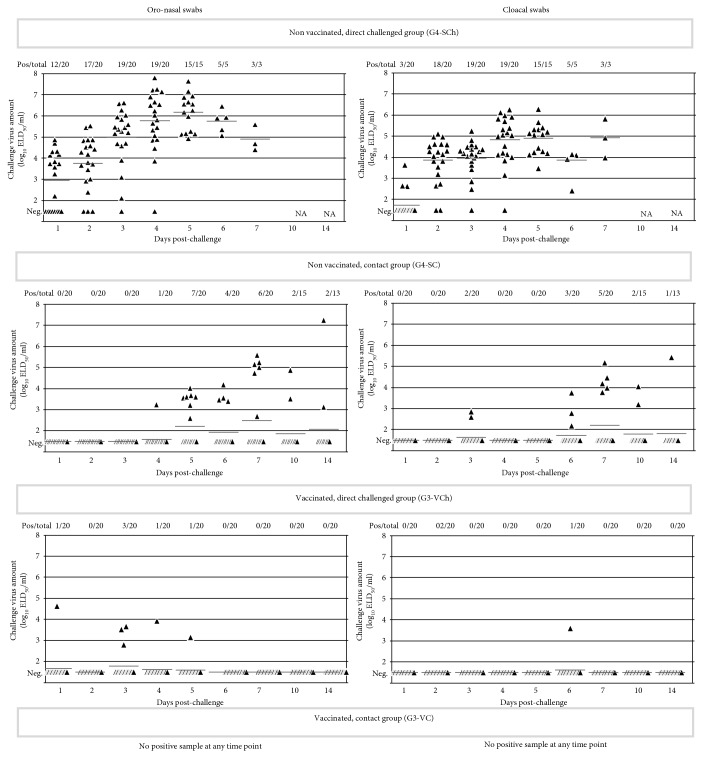
Oronasal and cloacal shedding by layers. AIV load of swab samples measured by qRRT-PCR is presented as log_10_ELD_50_/ml in the scatterplot; horizontal bars represent group mean value for each date. For calculation of mean, negative samples were given a value of 1.5 log_10_ ELD_50_/ml. Contact challenged groups (G3-VC and G4-SC) were comingled with the direct-challenged groups (G3-VCh and G4-SCh) from 8 hours postchallenge. Sampling date refers to the time after direct challenge.

Oronasal virus shedding by the unvaccinated direct-challenged birds was already high from the first day pch. and continued to be high until the day they died. Shedding of virus by the contact birds started on the 4th day pch. in 5% of the birds, which increased to 35% by the next day, but 60% of the birds survived till the end of the observation period without shedding virus in their oronasal swabs (see Figure 6). AIV load in the samples collected from affected contacts was similarly high as in the ones collected from the direct-challenged animals (see Figure 6).

Cloacal shedding by the nonvaccinated direct-challenged chickens started already on the 1^st^ day pch. in 15% of the birds, increased rapidly, and continued until the day when the last bird died. Virus load in cloacal swabs was lower compared to oronasal swabs (see Figure 6). Delayed spreading of challenge virus to contact birds was observed, and the ratio of birds with detectable shedding at any date during the 14 days pch. observation period reached only 40% (see Figure 6).

Oronasal virus shedding by the vaccinated and direct-challenged chickens could be detected only in a small proportion (15%) of the birds and only at the first 5 days pch., while no virus could be detected in the ON swab samples of vaccinated contact chickens (see Figure 6).

Cloacal shedding of challenge virus by the direct-challenged vaccinated chickens was almost totally absent during the whole postchallenge observation period; only one chicken had moderate level of virus load in its cloacal swab at day 6 pch. Vaccinated contact chickens did not shed any detectable virus through the cloaca.

### 3.6. Reproduction Ratio (R)

Length of infectious period, transmission rate, and reproduction ratio values in the unvaccinated groups are summarized in Table 2. The estimate of the reproduction ratio in the unvaccinated broiler chickens is 1.84 (95% confidence interval: 1.11, 3.06), which is significantly above 1. It means that the virus can spread easily in an unvaccinated population of broiler chickens. The estimate of *R* is 0.69 (0.33, 1.44) for the unvaccinated layers. As the confidence interval for the reproduction ratio covers 1, we cannot reject either that *R* < 1 or *R* > 1.

Vaccinated contact chickens showed lack of challenge virus spread; therefore, the *R* value for the vaccinated groups is 0.00 regardless the type of chicken (i.e., broiler or layer).

### 3.7. Humoral Immune Response to Challenge

**Figure 7:**
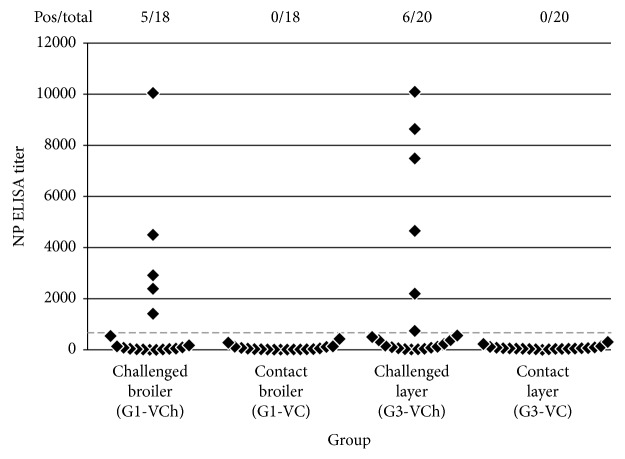
Presence of humoral immune response to challenge in vaccinated chickens was checked with commercial ELISA measuring antibodies against the NP protein. Positive results with ID Screen® Influenza A Nucleoprotein Indirect ELISA kit are above 668 titer (positivity limit is shown with dotted line).

Only a small proportion of the vaccinated direct-challenged birds developed antibody response to the NP of AIV in both the broiler and layer experiment (5 out of 18 in the broiler and 6 out of 20 in the layer experiment, see Figure 7), while the contact birds remained negative for NP antibodies. In the layer experiment, the survived unvaccinated contact chickens had no detectable antibody against the NP of AIV indicating the lack of virus transmission during the observed period.

## 4. Discussion

In poultry, vaccination against highly pathogenic avian influenza is not common; however, a number of countries (i.e., China, Hong Kong SAR, Vietnam, Indonesia, Bangladesh, South Korea, Pakistan, and Egypt) have used or continue to use vaccination in their fight against H5N1 avian influenza. The main objection against vaccination is that athough it provides clinical protection, it appears to be poorly effective in protecting against infection and controlling virus transmission; therefore, new infections can take place constantly without noticing [31].

We have used transmission experiments to study the efficacy of a rHVT-H5 vaccine-induced immunity on the transmission dynamics of HPAIV H5N8 strain both in commercial broilers and layers without MDA to AIV. In the unvaccinated groups, all direct-challenged birds became infected and died, which is in agreement with the highly pathogenic nature of the challenge virus. The mortality of the contact animals in the unvaccinated broiler group was also high (100%), indicating high transmission rate among unvaccinated individuals. Interestingly, only 40% of contacts in the unvaccinated layer group died and 45% got infected, indicating a less effective transmission of the virus to and among the contacts. More efficient spread of virus among broilers is reflected by the higher *R* value (1.84) compared to the one (0.69) in layers. High level of clinical protection (90% in broilers and 100% in layers) and very limited shedding followed by partial seroconversion to challenge was found in the direct-challenged vaccinated chickens, while the vaccinated contacts proved to be fully protected against infection (*R* value = 0.00). These results showed that information on morbidity and mortality, and quantification of virus shedding by the directly infected birds, as the regular method for the evaluation of vaccine efficacy, does not necessarily translate to transmission. Therefore, appropriate transmission experiments are needed that would allow to measure differences in infectivity of virus strains for different poultry species and breeds and to provide information on whether a vaccine is not only able to protect animals from morbidity and mortality but also to stop transmission with good effectiveness.

Other research groups that have tested the efficacy of this rHVT-H5 vaccine against clade 2.3.4.4 H5Nx viruses from Europe and the United States obtained also excellent cross protection (i.e., 90% or above [18, 20]) with the exception of a study performed with two US isolates from 2014, in which only 60% protection was found [19]. However, in that study [19], the prechallenge HI test of the vaccinated chickens (SPF) using an HA antigen homologous to the vaccine indicated only 70% seroconversion (7/10) with a range of HI titers from 5 to 7 log_2_, while the other 3 chickens were completely negative (1 log_2_ HI titer) at 4 weeks after vaccination. In the other published studies using the same rHVT-H5 vaccine in SPF chickens or broilers without MDA to AIV, more uniform seroconversion was reported at 4 weeks postvaccination with no or rare negative HI titers [16, 18, 20]. This may indicate that the birds showing no seroconversion might have missed vaccination; therefore, it is very advisable to monitor vaccination efficiency in the field by checking “vaccine take” with the detection of vaccine virus by qPCR from the feather pulp of randomly selected birds between 2 and 5 weeks of age.

It is the general assumption that the closer the antigenic similarity between vaccine and field strain is, the better the vaccine efficacy is expected to be. Failure of vaccination to prevent infection and transmission of HPAIV strains in the field is usually attributed to the antigenic distances between the inactivated vaccine and the circulating field strains and called for constant vaccine updating [32–34]. Our results reported here and the ones reviewed previously [17, 35], however, showed that rHVT-H5 vaccine could raise effective level of immunity against an antigenically distant virus.

Our results are in agreement with the findings, reported previously by others, that a sufficient level of host immunity induced by a vaccine can compensate for the antigenic difference between vaccine and field strain [36–40]. Immunity against avian influenza is largely based on the presence of antibodies against the surface proteins (NA and HA), from which the antibodies to HA are being far the most important [41]; therefore, this protein is expressed by most of the recombinant vaccines. Although the humoral immunity against HA protein is strong, its protective value is very strongly influenced by the difference between the inactivated vaccine strain and the challenge strain [6, 35]. In poultry, parenterally applied inactivated vaccines are widely used. In this case, humoral immune response to other cross-reactive proteins (e.g., transmembrane protein M2) is weak, which cannot compensate the effect of antigenic difference in HA [41].

Immunological background of cross protection against heterologous influenza infections has been investigated dominantly in mice and humans; only little information is available in chickens. Studies demonstrated that protection can occur in the absence or with very low level of serum HI antibodies against the challenge virus [18, 42], indicating that T cell-mediated immunity (CMI) plays a pivotal role in cross protection against drifted and heterologous strains [42–45]. Most of the studies on CMI focused on the conserved epitopes on internal proteins (e.g., NP and M), but it was revealed that HA specific T cell response can be detected after influenza vaccination or infection [46–48]. In chickens, a T cell epitope on AIV H5 HA molecule was identified by Haghighi et al. [27], which can be recognized by both CD4+ and CD8+ cells. This region showed only a single amino acid difference between the vaccine and the challenge virus used in our study, contrary to the presence of several amino acid differences in the other epitopes (see Figure 1). Clearance of influenza virus in the lungs of vaccinated mice was associated with significant CD4+ and CD8+ cell infiltration after heterologous challenge [42]. While CD8+ effector T cells kill the infected cells through their cytotoxic activity, CD4+ cells have a more diverse role mediating the maturing of CD8+ T cells, B cells, and the cytotoxic response [49]. Residence of memory CD4+ T cells in the lung afforded high level of protection in mice [50] and proved to be long lived with an enhanced capacity to protect against reinfection, due to their ability to respond rapidly and robustly [49]. On the contrary, Seo et al. found that the presence of memory CD8+ T cells expressing *γ*IFN is the key for cross protection in chickens [51]. B cells and the B cell-derived soluble factors also contribute to the effector CD8+ T cell function [42]. Internal viral proteins contain B cell epitopes that are conserved among influenza viruses, and the mucosal IgA has broader specificity than serum IgM [52] which both may contribute to the control of heterologous influenza infection.

Efficacy studies conducted with the rHVT-H5 vaccine focused on humoral immune response to vaccination, clinical protection, and shedding reduction, but some of them addressed the presence of CMI. Rauw et al. [16] showed CMI by the ChIFN*γ* production after ex vivo antigenic recall activation of lymphocytes from the spleen in broilers at 3 and 4 weeks of age, while Kapczynski et al. [53] demonstrated the presence of cross-reactive cytotoxic lymphocytes in the spleen of 4-week-old SPF chickens which were vaccinated with the rHVT-H5 vaccine at day old. Based on the reports on the induction of both humoral and cell-mediated immune response against AIV after rHVT-H5 vaccination in chickens and the more extensive studies in mammals on the immunological background of cross-reactivity between antigenically distant influenza viruses, it is likely that CMI accounts for the good immunity even when antibody titers to the challenge virus strain are low.

Although a classical vaccination-challenge type of experiment can provide information on whether a vaccine is able to provide clinical protection and allow quantification of virus shedding, it does not bring the solid information on effectiveness of vaccine to control transmission. To the best of our knowledge, only few experimental studies exist where transmission was evaluated in birds vaccinated with a strain being antigenically distant from the challenge strain [35, 54–56]. Our results suggest that it is important to ascertain whether a vaccine selected to be used in a vaccination campaign is able to stop transmission by estimating the transmission magnitude of circulating field strains in vaccinated animals. Therefore, the results of our study further highlight the needs for carrying out appropriate transmission experiments that would allow the evaluation of any AI vaccine to be used in the control of HPAI for the effectiveness to stop transmission, which is one of the most important aims of vaccination, especially with regard to containing an epidemic.

Our study results offer evidence that rHVT- H5 vaccine could protect animals from infection and transmission, even if remarkable antigenic distance between vaccine and challenge strains exists. Using this type of vaccine in the prevention and control of HPAI could be attractive since the constant vaccine updating, required for inactivated whole virus antigen vaccines, could be reduced or eliminated. Apart from using a proper vaccine, it is equally important to make certain that the expected level of vaccination coverage is reached, and as a result, proper population immunity can be expected, which could be best accomplished when vaccination is done in the hatchery under controlled conditions, for which the use of HVT-based recombinant vaccine is well suited. If vaccination is not done properly, it will fail to elicit adequate level of herd immunity, which will in turn lead to insufficient protection against infection.

## Figures and Tables

**Table 1:** Summary of the two transmission experiments set up.

Type of chickens	Group	Vaccination	Subgroup	Challenge	Abbreviation
Broiler chickens	Group 1	rHVT-H5 vaccine at hatch, s.c.	Vaccinated challenged	Direct challenge at 5 weeks of age	G1- VCh
			Vaccinated contact	Physical contact from 8 h postchallenge	G1- VC
	Group 2	No	Susceptible challenged	Direct challenge at 5 weeks of age	G2- SCh
			Susceptible contact	Physical contact from 8 h postchallenge	G2- SC

Layer chickens	Group 3	rHVT-H5 vaccine at hatch, s.c.	Vaccinated challenged	Direct challenge at 7 weeks of age	G3- VCh
			Vaccinated contact	Physical contact from 8 h postchallenge	G3-VC
	Group 4	No	Susceptible challenged	Direct challenge at 7 weeks of age	G4-SCh
			Susceptible contact	Physical contact from 8 h postchallenge	G4- SC

**Table 2:** Overview of the statistical analyses (95% CI in brackets).

Group	Infectious period (day)		Transmission rate (*β*, 1/day)	Reproduction ratio (*R*)
	Challenged group	Contact group		
Unvaccinated broilers	3.55 (3.11–3.99)	3.42 (2.68–4.16)	0.54 (0.34–0.85)	1.84 (1.11–3.06)
Unvaccinated layers	4.55 (4.19–4.91)	4.70 (2.73–6.67)	0.15 (0.08–0.27)	0.69 (0.33–1.44)

## Data Availability

The data used to support the findings of this study are available from the corresponding author upon request.
